# Polymorphism in the major histocompatibility complex (MHC class II B) genes of the Rufous-backed Bunting (*Emberiza jankowskii*)

**DOI:** 10.7717/peerj.2917

**Published:** 2017-01-25

**Authors:** Dan Li, Keping Sun, Yunjiao Zhao, Aiqing Lin, Shi Li, Yunlei Jiang, Jiang Feng

**Affiliations:** 1 Jilin Provincial Key Laboratory of Animal Resource Conservation and Utilization, Northeast Normal University, Changchun, China; 2 College of Animal Science and Technology, Jilin Agricultural University, Changchun, China

**Keywords:** *Emberiza jankowskii*, *Emberiza cioides*, MHC, Positive selection, Trans-species polymorphism

## Abstract

Genetic diversity is one of the pillars of conservation biology research. High genetic diversity and abundant genetic variation in an organism may be suggestive of capacity to adapt to various environmental changes. The major histocompatibility complex (MHC) is known to be highly polymorphic and plays an important role in immune function. It is also considered an ideal model system to investigate genetic diversity in wildlife populations. The Rufous-backed Bunting (*Emberiza jankowskii*) is an endangered species that has experienced a sharp decline in both population and habitat size. Many historically significant populations are no longer present in previously populated regions, with only three breeding populations present in Inner Mongolia (i.e., the Aolunhua, Gahaitu and Lubei557 populations). Efforts focused on facilitating the conservation of the Rufous-backed Bunting (*Emberiza jankowskii*) are becoming increasingly important. However, the genetic diversity of *E. jankowskii* has not been investigated. In the present study, polymorphism in exon 2 of the MHCIIB of *E. jankowskii* was investigated. This polymorphism was subsequently compared with a related species, the Meadow Bunting (*Emberiza cioides*). A total of 1.59 alleles/individual were detected in *E. jankowskii* and 1.73 alleles/individual were identified in *E*. *cioides*. The maximum number of alleles per individual from the three *E. jankowskii* populations suggest the existence of at least three functional loci, while the maximum number of alleles per individual from the three *E. cioides* populations suggest the presence of at least four functional loci. Two of the alleles were shared between the *E. jankowskii* and *E. cioides*. Among the 12 unique alleles identified in *E. jankowskii*, 10.17 segregating sites per allele were detected, and the nucleotide diversity was 0.1865. Among the 17 unique alleles identified in *E. cioides*, eight segregating sites per allele were detected, and the nucleotide diversity was 0.1667. Overall, compared to other passerine birds, a relatively low level of MHC polymorphism was revealed in *E. jankowskii*, which was similar to that in *E. cioides*. Positive selection was detected by PAML/SLAC/FEL analyses in the region encoding the peptide-binding region in both species, and no recombination was detected. Phylogenetic analysis showed that the alleles from *E. jankowskii* and *E. cioides* belong to the same clade and the two species shared similar alleles, suggesting the occurrence of a trans-species polymorphism between the two *Emberiza* species.

## Introduction

Some natural populations are currently experiencing a significant decrease in available habitats as well as fragmentation of existing habitats. These factors have culminated in a dramatic decline in population sizes (Wahlberg, Moilanen & Hanski, 1996; Peacock & Smith, 1997). Small populations often display low genetic diversity due to genetic drift and purging of deleterious alleles during inbreeding (Harrison & Hastings, 1996; Keller & Waller, 2002), ultimately leading to local and global extinction of species (Lande & Shannon, 1996; Hedrick & Kalinowski, 2000; Dlugosch & Parker, 2008). Therefore, investigating the genetic diversity of endangered species is essential for species conservation.

The major histocompatibility complex (MHC) genes play an important role in ecological adaptation. It is possible that these adaptive genes play an important role in maintaining variation in severely reduced population sizes (Robertson, 1962; Oliver & Piertney, 2012), which have been utilized to investigate the genetic diversity of wildlife populations (Bollmer et al., 2011; Vásquez-Carrillo et al., 2014). MHC genes are known to be highly polymorphic and play a major role in immune function. MHC class I molecules present peptides from intracellular pathogens, whereas MHC class II molecules display peptides from extracellular pathogens (Piertney & Oliver, 2006). The peptide-binding region (PBR) encoded by exon 2 of MHCIIB is a highly polymorphic region in vertebrates (Anmarkrud et al., 2010; Eimes et al., 2015). This region presents peptides to T-lymphocytes, which in turn induces an immune response to non-self peptides (Piertney & Oliver, 2006).

Pathogen-mediated selection is the driving force behind the genetic diversity associated with MHC gene loci (Bernatchez & Landry, 2003), and some studies have suggested that gene conversion, recombination, or sexual selection may play an important role in maintaining MHC genetic diversity (Promerová et al., 2013; Sardell, Kempenaers & Duval, 2014). In a wide range of taxa, trans-species polymorphisms (TSPs) may contribute to MHC genetic diversity (Ballingall et al., 2010; Luo & Pan, 2013; Jaratlerdsiri et al., 2014; Eimes et al., 2015; Balasubramaniam et al., 2016). TSP occurs when alleles are shared between species as a result of orthologous MHC allelic lineages being maintained by balancing selection and persisting through speciation events (Klein et al., 1998; Lenz et al., 2013). However, it is also possible that similar alleles arise through convergent evolution (Eimes et al., 2015; Balasubramaniam et al., 2016). In general, convergence and TSP can be examined by constructing phylogenetic trees and comparing clustering patterns of trees reconstructed from non-synonymous substitutions at codons likely to be under selection with trees reconstructed from synonymous substitutions at putatively neutral sites (Graser et al., 1996; Kupfermann et al., 1999; Kriener et al., 2000; Li, Zhou & Chen, 2011). If alleles are more similar at neutral sites than at putatively selected sites between species, TSP is indicated. Conversely, if alleles are more similar at putatively selected sites compared to neutral sites between species then convergent evolution is more likely (Klein et al., 1998). When a population adapts to its new environment, MHC allele diversity is comprised of alleles maintained through TSP and new genetic variants.

The Rufous-backed Bunting (*Emberiza jankowskii*) is a small passerine bird (Passeriformes, Emberizidae) that has no described phenotypic variation. It is listed as an endangered species by the International Union for Conservation of Nature and Natural Resources (BirdLife International, 2010). The species has been subjected to a sharp decline in population size as well as habitat fragmentation (Wang, Jiang & Gao, 2010). In order to prevent extinction, an understanding of the current genetic diversity of endangered species is important. Additionally, because of the dearth of information pertaining to genetic diversity of *E. jankowskii*, this study was undertaken to investigate polymorphisms in the exon 2 of the MHCIIB of *E. jankowskii.* A related species, the Meadow Bunting (*Emberiza cioides*), was used for comparison.

Historically, two *E. jankowskii* breeding populations (eastern and western) have been recognized. The eastern breeding populations occur southeast of Heilongjiang Province, east of Jilin Province, to the extreme south of Russian Far East and the boundary region between Russia, China, and North Korea (Yamashina, 1957; Fu & Chen, 1966; Panov, 1973; Stresemann & Portenko, 1981; Zhao, Nickel & Groh, 1994). The western populations occur in west Jilin Province and the adjacent areas of Inner Mongolia (Fu & Chen, 1966; Zhao, Nickel & Groh, 1994; Gao, 2002). However, no information pertaining to either of these two breeding distributions has been reported in recent years (Wang, Jiang & Gao, 2010). The recently discovered breeding populations (Aolunhua (ALH), Gahaitu (GHT) and Lubei557 (LB)) are distributed in Inner Mongolia and belong to the western breeding population of *E. jankowskii*. We only observed these three populations between May and August 2012. *Emberiza jankowskii* is phenotypically similar to a more common species, *E. cioides*. Recent studies have suggested that *E. jankowskii* and *E. cioides* have a close genetic relationship and always cluster into the same highly supported clade with two other congeneric species, based on analyses of mitochondrial (*Cytb*, *Cox gene*) and nuclear (*ODC gene*, *AFLP*) loci (Alström et al., 2008; Liang et al., 2008; Li et al., 2013). Compared with the limited distribution of *E. jankowskii*, *E. cioides* populations are more frequently observed. Therefore, these two passerine birds represent good relative models to investigate the genetic diversity of MHC genes and associated genetic maintenance mechanisms in *E. jankowskii*. Two sympatrically distributed and one non-sympatrically distributed *E. cioides* populations were chosen for comparison with *E. jankowskii*. In general, common species are more genetically diverse than endangered species (Akst, Boersma & Fleischer, 2002; Bollmer, Vargas & Parker, 2007). In this study, we investigated polymorphisms in the exon 2 of the MHCIIB gene of both *E. jankowskii* and *E. cioides*. The aims of this study were to: (i) reveal the genetic diversity of the MHCIIB gene in *E. jankowskii* and *E. cioides*; (ii) screen for associated selection and recombination signatures; (iii) determine the extent of TSP in *E. jankowskii*; and (iv) aid in the proposal of measures to protect *E. jankowskii*.

## Materials & Methods

### Ethics statement

All field studies were approved by the National Animal Research Authority in Northeast Normal University, China (approval number: NENU-20080416). The blood samples (up to 10 µl) were extracted from birds by wing vein puncture. The birds were subsequently released.

### Samples

**Figure 1:**
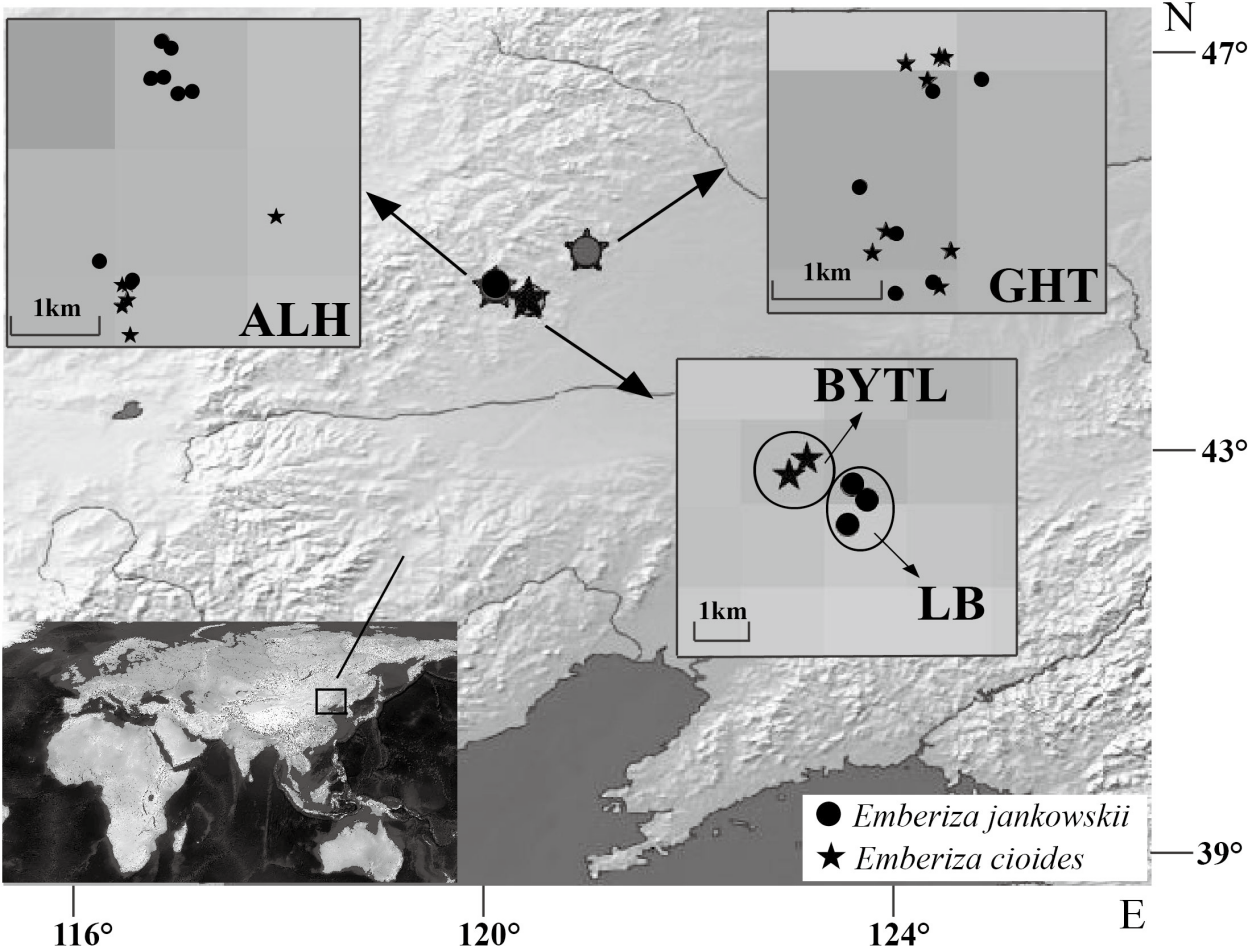
Sample distribution of *Emberiza jankowskii* and *Emberiza cioides*. GHT represents Gahaitu, LB represents Lubei557, ALH represents Aolunhua, and BYTL represents Bayantala. The sample distribution map was generated with QGIS 2.16 (http://www.qgis.org) and Natural Earth public domain map data (http://www.naturalearthdata.com/about/terms-of-use/), and modified in Adobe Illustrator.

Blood samples from 17 *E. jankowskii* individuals were collected from three populations: ALH, GHT and LB. Blood samples from 15 *E. cioides* individuals were also collected from three locations: ALH, GHT and Bayantala (BYTL) (Fig. 1; Table 1). The blood samples (up to 10 µl) were collected by wing vein puncture. They were subsequently mixed with 1 ml of absolute ethyl alcohol and stored at room temperature. Sampling sites and the number of individuals that were sampled are presented in Table 1 and Fig. 1.

**Table 1:** Population allele distributions and MHC variation in *Emberiza jankowskii* and *Emberiza cioides*.

Species	Population	*N*	Number of alleles	Mean number of alleles per individual	Maximal number of alleles in one sample	Minimal number of loci	Number of variable sites (*S*)	Nucleotide diversity (*π*)
*Emberiza jankowskii*	ALH	8	11	1.38	5	3	122	0.1913
	GHT	6	10	1.67	5	3	105	0.1835
	LB	3	6	2	4	2	101	0.1980
	All	17	27	1.59	5	3	122	0.1865
*Emberiza cioides*	ALH	5	8	1.60	5	3	122	0.2114
	GHT	8	11	1.38	5	3	112	0.1440
	BYTL	2	7	3.50	7	4	98	0.1591
	All	15	26	1.73	7	4	136	0.1667

**Notes.**

GHT represents Gahaitu LB represents Lubei557 ALH represents Aolunhua BYTL represents Bayantala

### DNA extraction and amplification

DNA was extracted from blood samples using the AxyPrep™ Blood Genomic DNA Miniprep Kit (Axygen, Hangzhou, China), following the manufacturer’s protocol. Exon 2 of the MHCIIB gene was amplified by polymerase chain reaction (PCR) using standard procedures and Premix Taq™ (TaKaRa Taq™ Version 2.0 plus dye) (TaKaRa, Dalian, China). The PCR was performed in a 50-µl mixture consisting of 1 µl of genomic DNA (approximately 50 ng), 1 µl of the respective primers, Int1f.7 and Int2r.1 (10 µmol/l) (Edwards, Gasper & March, 1998; Aguilar et al., 2006), 25 µl of Premix Taq™ (TaKaRa Taq™ Version 2.0 plus dye) (TaKaRa, Dalian, China), and 22 µl of ddH_2_O. The reaction was performed in a 2027 Thermal Cycler (Applied Biosystems) at 94 °C for 5 min, 30 cycles of 94 °C for 30 s, 59 °C for 30 s, and 72 °C for 30 s, and a final extension step at 72 °C for 10 min. The PCR products were separated using 1% agarose gel electrophoresis. The primers targeted a 485–503 bp fragment that included a segment of intron 1 (202–220 bp), the entire sequence associated with exon 2 (270 bp), and a segment of intron 2 (13 bp).

### Cloning and sequencing

The PCR products were purified using a SanPrep column PCR product purification kit (Sangon, Shanghai, China) and ligated into the pMD^®^18-T Vector (TaKaRa, Dalian, China). The recombinant vector was then transformed into *Escherichia coli* DH5*α* chemically competent cells as recommended by the supplier (TaKaRa, Dalian, China). The transformed cells were grown at 37 °C on LB agar supplemented with ampicillin (100 µg ml^−1^). Positive clones were picked out using a sterile toothpick. The associated colonies were diluted in 6 µl of ddH_2_O. The dilutions were used directly as DNA templates in the PCR reactions. The clones were amplified using the PCR conditions outlined in the “DNA extraction and amplification” section. Standard M13-primers were utilized to facilitate amplification. Approximately 20 positive clones per individual were sequenced by Come (Comate Bioscience Co. Ltd., Changchun, China).

### Data analyses

Nucleotide sequences were aligned using BioEdit (Hall, 1999). DNA sequences were confirmed as MHCIIB exon 2 following the use of BLAST searches with GenBank. To avoid false haplotypes, only sequences detected in two or more individuals were included in the analysis (Lenz & Becker, 2008; Nadachowska-Brzyska et al., 2012). The number of alleles, segregating sites (S) and nucleotide diversity (*π*) in each population were calculated using DnaSP 5.0 (Librado & Rozas, 2009).

The sequences from each population were treated as one for each species in the tests below. The putative PBR in *E. jankowskii* and *E. cioides* was assigned according to the PBR described by Tong et al. (2006). The identification of sites that were subject to selection in the MHC alleles was performed using six methods. First, standard selection tests (Tajima’s D, Fu & Li’s F* and Fu & Li’s D*) were performed using DnaSP 5.0 (Librado & Rozas, 2009). Second, the non-synonymous (d_*N*_) and synonymous (d_*S*_) substitution ratio (*ω* = d_*N*_/d_*S*_) was used to measure selection pressure at the amino acid level (Nei & Kumar, 2000; Yang & Nielsen, 2002). The parameter *ω* was calculated with MEGA v6.0 (Tamura et al., 2013) using the method described by Nei & Gojobori (1986), with Jukes Cantor corrections and 1,000 bootstrap replicates. The *Z*-test (Nei & Gojobori, 1986) was used to determine the probability of selection by comparing the selection parameter, *ω*, against a null hypothesis of strict neutrality (d_*N*_ = d_*S*_). Third, synonymous substitution rates can be affected by highly biased codon usage (Yang & Bielawski, 2000), thus the d_*N*_/d_*S*_ ratio was calculated using the YN00 method accounting for codon-usage bias of Yang & Nielsen (2000) implemented in the Phylogenetic Analysis by Maximum Likelihood package (PAML 4.8) (Yang, Wong & Nielsen, 2005; Yang, 2007). A selectively neutral gene is represented by the equation, *ω* = 1, whereas a gene undergoing positive selection shows *ω* >1.

The fourth method involved the identification of sites that were subject to selection using the maximum likelihood method as implemented in CODEML in PAML 4.8. We tested the M1a (nearly neutral), M2a (positive selection), M7 (beta), and M8 (*β* and *ω*) models for codon substitutions. A variation in the *ω* ratio was permitted among the sites investigated. In the analysis, a likelihood ratio test for positive selection was performed by comparing model M1a against M2a, and M7 against M8. *P*-values were calculated using a chi-squared test. For each *Emberiza* species, we also used additional two methods of single-likelihood ancestor counting (SLAC) and fixed-effects likelihood (FEL) in HyPhy package (Kosakovsky Pond & Frost, 2005) implemented in the Datamonkey web server (Delport et al., 2010; www.datamonkey.org) to identify the individual codons under selection. We used the genetic algorithm recombination detection (GARD; Kosakovsky Pond et al., 2006) method to rule out recombination as a confounding effect before performing the SLAC/FEL analyses and considered only those amino acids that were identified as being under selection by at least one likelihood method. Also, we matched the position of codons under selection in our study to human (Tong et al., 2006) and other avian species (Anmarkrud et al., 2010; Zagalska-Neubauer et al., 2010; Sutton et al., 2013; Borg et al., 2011).

We constructed nucleotide phylogenies from three different partitions of exon 2: non-synonymous sites identified using the putative PBR sites (45 bp), putative non-PBR sites (222 bp) inferred from HLA and the contiguous 270 bp fragment of exon 2. For the former two partitions, we constructed the unrooted trees using a neighbor-joining (NJ) method in MEGA. For the analysis of the contiguous 270 bp fragment, the HLA (GenBank Accession No.: NM_021983) and the GAME from the Great Snipe (*Gallinago media*) (GenBank Accession No.: AF485413) genes were used as outgroups, and the phylogenetic relationship of exon 2 alleles of the MHCIIB gene of *E. jankowskii* and *E. cioides* was examined by constructing a neighbor-joining (NJ) tree using MEGA and a Bayesian inference (BI) tree using MrBayes v3.1.2 (Ronquist & Huelsenbeck, 2003). The NJ analysis was underpinned by the assumption that homogenous substitution patterns among lineages existed, along with the occurrence of uniform rates among analyzed sites. A consensus tree was generated from 10,000 bootstrap replicates using MEGA. For the Bayesian analysis, an optimal model of nucleotide substitution was selected using the Akaike Information Criterion (AIC) (Akaike, 1974) as determined in jModelTest (Posada, 2008; Posada, 2009) for each independent codon position (Position 1: TIM3ef + I + G; Position 2: TVM + G; Position 3: TPM2uf + G). Two independent parallel runs of four incrementally heated Metropolis-coupled Monte Carlo Markov chains (MCMCs) were conducted, with trees sampled every 10 generations for 1,000,000 generations. The analyses were deemed to have converged when the average standard deviation of split frequencies was <0.1. The first 25% of the generations were discarded as “burn-in.”

For each species separately and both species pooled, the RDP3 Alpha 44 (Heath et al., 2006) package was used to simultaneously test for signatures of recombination using multiple algorithms, including RDP (Martin & Rybicki, 2000), GENECONV (Padidam, Sawyer & Fauquet, 1999), BootScan (Martin et al., 2005), MaxChi (Smith, 1992), Chimaera (Posada & Crandall, 2001), Siscan (Gibbs, Armstrong & Gibbs, 2000), and 3Seq (Boni, Posada & Feldman, 2007). To minimize a false-positive error rate, the highest acceptable *P* value for inferring recombination events was set at 0.000005, with a window size of 20 nucleotides, and only those breakpoints that were identified using at least four methods were considered valid.

**Figure 2:**
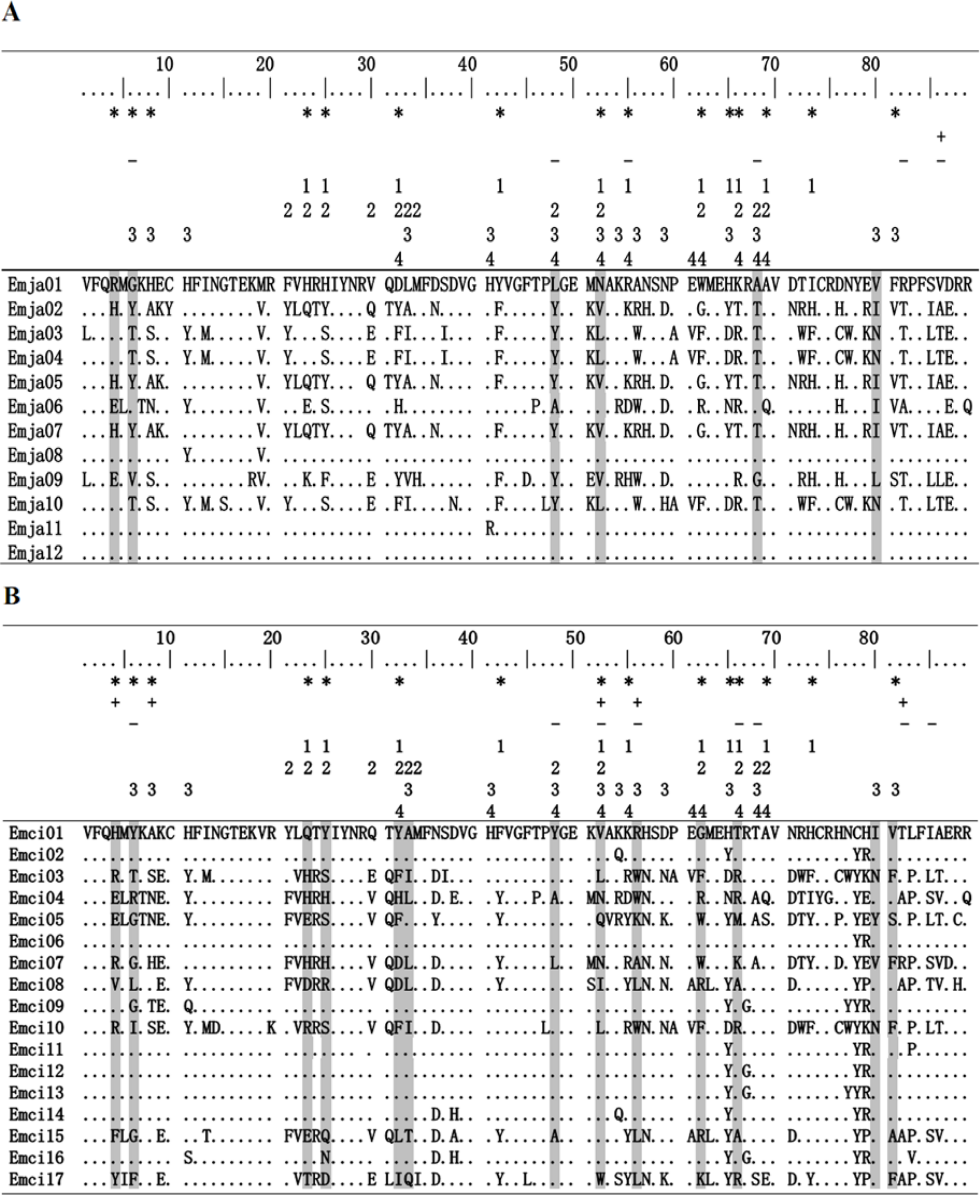
Alignment of MHCIIB exon 2 amino acid sequences. *Emberiza jankowskii* and *Emberiza cioides* were the two species selected for this analysis. Periods and dots indicate identity with the Emja01 or Emci01 sequence. Emja04 = Emci03, Emja05 = Emja07 = Emci11, Emja01 = Emja12. Emja represents *Emberiza jankowskii*, Emci represents *Emberiza cioides*. * represents putative peptide-binding sites based on Tong et al. (2006); + represents sites identified by SLAC in this study; **-** represents sites identified by FEL in this study; **1**represents *Luscinia svecica* (FJ529861; Anmarkrud et al., 2010); **2** represents *Ficedula albicollis* (HQ678311; Zagalska-Neubauer et al., 2010); **3** represents *Philesturnus carunculatu*s (KF225737; Sutton et al., 2013); **4** represents *Passer domesticus* (Pado-DAB*301; Borg et al., 2011). Sites identified by CODEML as being under positive selection by model M8 are shaded gray.

## Results

### Nucleotide diversity

Exon 2 sequences from the MHCIIB gene that were 270 bp in length were amplified from 17 *E. jankowskii* individuals and 15 *E. cioides* individuals. A total of 554 sequences were obtained. Among these, 275 of the sequences were derived from the *E. jankowskii* individuals, while 279 of the sequences came from *E. cioides* individuals. Nucleotide sequences that translated into amino acid sequences lacking a frameshift mutation or stop codon were confirmed as functional alleles (Fig. 2). Twelve different alleles were confirmed in *E. jankowskii*. These alleles were designated as Emja01–12. Seventeen different alleles were confirmed in *E. cioides* and were identified as Emci01–17. The number of alleles per individual for *E. jankowskii* and *E. cioides* was 1.59 and 1.73, respectively. These sequences were deposited in GenBank under the accessions KT751182 –KT751210. Two alleles (Emci03 = Emja04 and Emci11 = Emja07) were shared between *E. jankowskii* and *E. cioides*. A maximum of five alleles in an *E. jankowskii* individual and seven alleles in an *E. cioides* individual were detected (Table 1). Assuming that all loci were heterozygous, the minimum number of MHCIIB loci was estimated to be three in *E. jankowskii* and four in *E. cioides.* Following characterization of all *E. jankowskii* alleles, 122 segregating sites (S) were identified with an associated nucleotide diversity (*π*) of 0.1865. Analysis of the *E.  cioides* alleles revealed that *S* = 136, and *π* = 0.1667. The number of segregating sites after correcting for the number of alleles was 10.17 in *E. jankowskii* and 8 in *E. cioides*.

The mean number of alleles per individual (alleles/ind) and nucleotide diversity (*π*) was different between the two species in the two sympatric *E. jankowskii* and *E. cioides* populations. In ALH, *E. jankowskii* displayed a lower number of alleles/ind (1.38 vs. 1.60) and *π* (0.1913 vs. 0.2114) compared with *E. cioides*; however, the number of alleles/ind (1.67 vs. 1.38) and *π* (0.1835 vs. 0.1440) in *E. jankowskii* was higher than that in *E. cioides* in GHT (Table 1).

### Selection and recombination

Traditional selection statistics did not reveal any statistically significant signal of selection that deviated from the neutral expectations for *E. jankowskii* (Tajima’s D = 0.03, *p* > 0.10; Fu & Li’s D* = 0.17, *p* > 0.10; Fu & Li’s F* = 0.15, *p* > 0.10) and *E. cioides*(Tajima’s D = −1.03, *p* > 0.10; Fu & Li’s D* = −0.82, *p* > 0.10; Fu & Li’s F* = −1.02, *p* > 0.10). However, the d_*N*_ value was higher than the d_*S*_ value at all sites in *E. jankowskii* and *E. cioides*(Table 2). The *Z*-test revealed significant *P* values at all sites for both species, apart from the putative PBR (*Z*-test, Z*d*_*N*_ − *d*_*S*_ = 0.270, *P* = 0.788) of *E. jankowskii* and the putative PBR (*Z*-test, Z*d*_*N*_ − *d*_*S*_ = 1.834, *P* = 0.069) of *E. cioides* populations (Table 2). Nevertheless, based on the method of Yang and Nielsen, the putative non-PBR sites generated d_*N*_/d_*S*_ of just 0.817 in *E. jankowskii* and 0.786 in *E. cioides*.

**Table 2:** Comparison of rates of synonymous (d_*S*_) and non-synonymous (d_*N*_) substitutions; the value of *Z*-test of positive selection and probability (*P*) that positive selection acts on these sites.

Species		d_*N*_ ± SE	d_*S*_ ± SE	d_*N*_/d_*S*_	*Z*-test	*p*
*Emberiza jankowskii*	putative PBR	0.846 ± 0.169	0.777 ± 0.172	1.089	0.270	0.788
	putative non-PBR	0.169 ± 0.027	0.089 ± 0.025	1.899	2.387	0.019
	all sites	0.239 ± 0.033	0.157 ± 0.031	1.522	2.128	0.035
*Emberiza cioides*	putative PBR	0.839 ± 0.139	0.467 ± 0.129	1.797	1.834	0.069
	putative non-PBR	0.151 ± 0.022	0.074 ± 0.016	2.041	3.813	0.0002
	all sites	0.219 ± 0.029	0.123 ± 0.023	1.780	3.638	0.0004

**Notes.**

Values of *p* < 0.05 are considered significant.

The application of the likelihood model using the PAML/SLAC/FEL software showed that exon 2 of the MHCIIB gene underwent positive selection (Tables 3 and 4). The models M2a and M8 that allow for positive selection fit our data significantly better than the null hypothesis models M1a and M7 (Table 3). In total, we detected 9 codons under positive selection using at least one test (PAML/SLAC/FEL) in *E. jankowskii*, of which 6 sites (66.67%) matched homologous codons under positive selection found in other avian species and only 4 (44.44%) matched human antigen binding sites (Fig. 2A; Tables  3 and 4). In *E. cioides*, 17 codons under positive selection were found using at least one test (PAML/SLAC/FEL), of which 14 sites (82.35%) matched homologous codons under positive selection found in other avian species and only 10 (58.82%) matched human antigen binding sites (Fig. 2B; Tables 3 and 4). No recombination was detected in the exon 2 of the MHCIIB gene in *E. jankowskii* or *E. cioides*.

**Table 3:** Parameter estimates and results from four selection models as implemented in CODEML.

Species	Comparison	Model	Log-likelihood	Parameter estimates	Positively selected sites
*Emberiza jankowskii*	Emja 01-12	M1a (nearly neutral)	−1069.028	*p*_0_ = 0.539, *p*1 = 0.461, *ω*0 = 0.115, *ω*1 = 1.000	Not allowed
		M2a (positive selection)	−1064.197	*p*_0_ = 0.506, *p*1 = 0.458, *p*2 = 0.036, *ω*0 = 0.122, *ω*1 = 1.000, *ω*2 = 7.595	6G^*^, 48L, 68A
		M7 (beta)	−1071.512	*p* = 0.341, *q* = 0.345	Not allowed
		M8 (beta and omega)	−1065.897	*p*_0_ = 0.948, *p*1 = 0.052, *p* = 0.355, *q* = 0.330, *ω* = 6.084	4R, 6G^**^, 48L, 52N, 68A, 80V
*Emberiza cioides*	Emci 01-17	M1a (nearly neutral)	−1571.846	*p*_0_ = 0.548, *p*1 = 0.452, *ω*0 = 0.086, *ω*1 = 1.000	Not allowed
		M2a (positive selection)	−1550.821	*p*_0_ = 0.493, *p*1 = 0.368, *p*2 = 0.138, *ω*0 = 0.103, *ω*1 = 1.000, *ω*2 = 4.775	4H^*^, 6Y^**^, 23Q^*^, 25Y, 32Y, 33A^*^, 48Y^**^, 52V^*^, 66T^**^, 80I, 81V
		M7 (beta)	−1577.640	*p* = 0.290, *q* = 0.349	Not allowed
		M8 (beta and omega)	−1554.625	*p*_0_ = 0.836, *p*1 = 0.164, *p* = 0.348, *q* = 0.427, *ω* = 4.246	4H^**^, 6Y^**^, 23Q^**^, 25Y, 32Y, 33A^**^, 48Y^**^, 52V^*^, 56R, 62G, 66T^**^, 80I, 81V

**Notes.**

Positively selected sites were identified in models M2a and M8 by the Bayes empirical Bayes procedure (Yang, Wong & Nielsen, 2005).

* indicates that the posterior probability is >95%.

** indicates that the probability is >99%.

Emja *Emberiza jankowskii* Emci * Emberiza cioides*

**Table 4:** Results from two likelihood models (after performing GARD analysis) to infer amino acids under positive selection.

Species	Comparison	Condon	SLAC, *d*_*N*_ −*d*_*S*_	SLAC, *p* value	FEL, *d*_*N*_ −*d*_*S*_	FEL, *p* value
*Emberiza jankowskii*	Emja01-12	6	—	—	9.677	0.050
		48	—	—	4.911	0.115
		55	—	—	6.444	0.230
		68	—	—	3.084	0.074
		82	—	—	3.152	0.164
		86	7.586	0.207	5.101	0.114
*Emberiza cioides*	Emci01-17	4	1.915	0.225	—	—
		6	—	—	17.230	0.010
		8	1.064	0.233	—	—
		30	—	—	—	—
		32	—	—	—	—
		33	—	—	—	—
		48	—	—	5.092	0.041
		52	5.166	0.240	9.161	0.155
		56	1.460	0.155	3.402	0.164
		66	—	—	4.531	0.122
		68	—	—	0.868	0.152
		82	0.918	0.201	2.040	0.093
		85	—	—	2.692	0.228

**Notes.**

Amino acid sites with *p* values ¡0.25 for SLAC and FEL were considered as to be under positive selection. The sign “—” represents no evidence for positive selection.

Emja *Emberiza jankowskii* Emci * Emberiza cioides*

**Figure 3:**
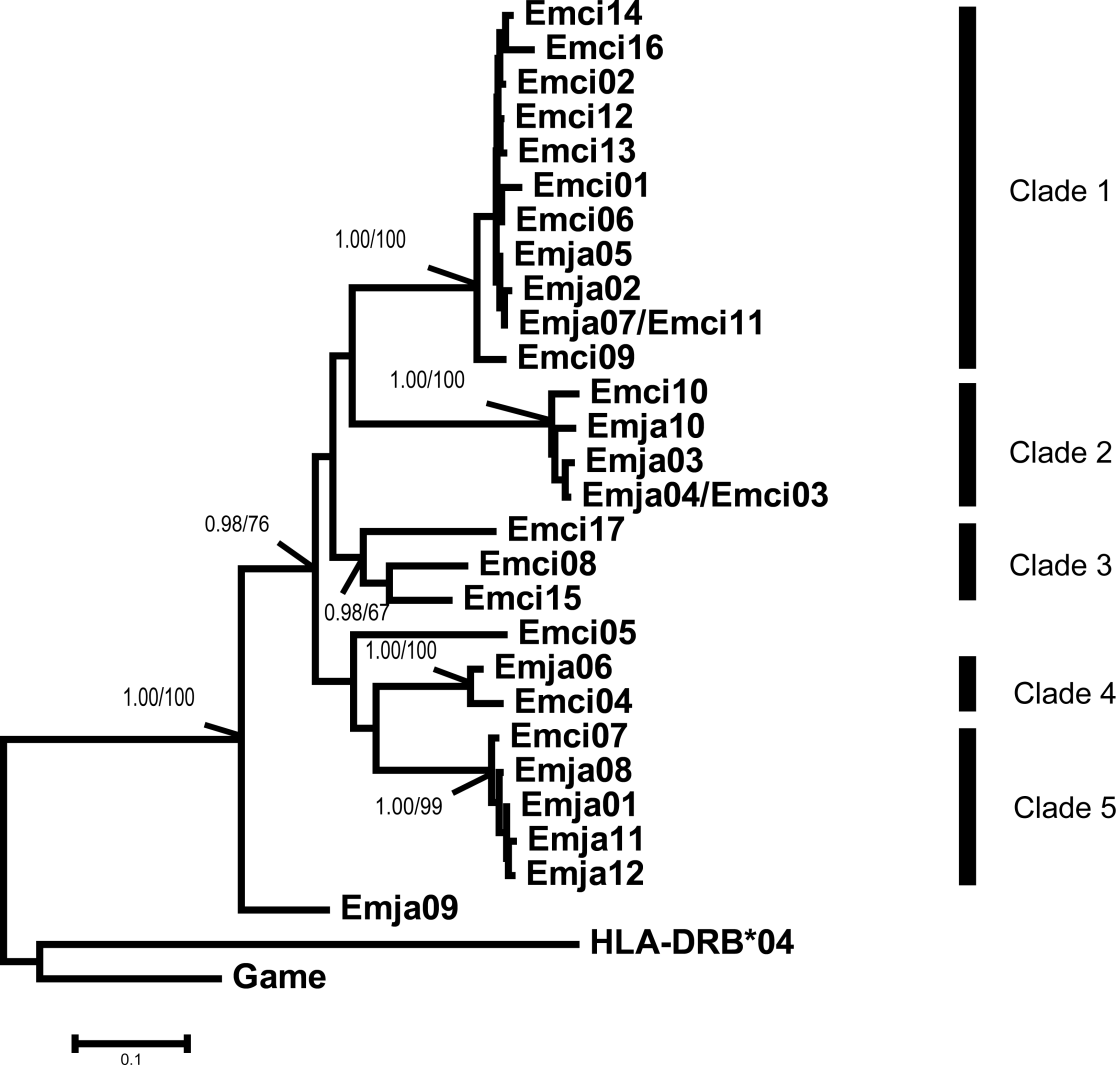
*Emberiza jankowskii* and *Emberiza cioides* MHCIIB exon 2 tree. HLA-DRB*04 is the human MHCIIB exon 2 allele (GenBank Accession No.: NM_021983), and Game is the *Gallinago media* MHCIIB exon 2 allele (GenBank Accession No.: AF485413); these were used as outgroups. The tree constructed by the contiguous 270 bp fragment of exon 2. All the clades were labeled, including bootstrap support and posterior probabilities (BI and NJ). Emja07/Emci11 and Emja04/Emci03 represent identical alleles from *Emberiza jankowskii* and *Emberiza cioides.* Emja, *Emberiza jankowskii*; Emci, * Emberiza cioides*.

### Phylogenetic analysis

The MHCIIB exon 2 sequences were clustered into five clades based on the results of Bayesian and neighbor-joining analyses (Fig. 3). The alleles of both species were represented in Clades 1, 2, 4 and 5, and all clades showed high bootstrap support values and posterior probabilities (BI and NJ). Clade 3 contained only alleles found in *E. cioides*.

**Figure 4:**
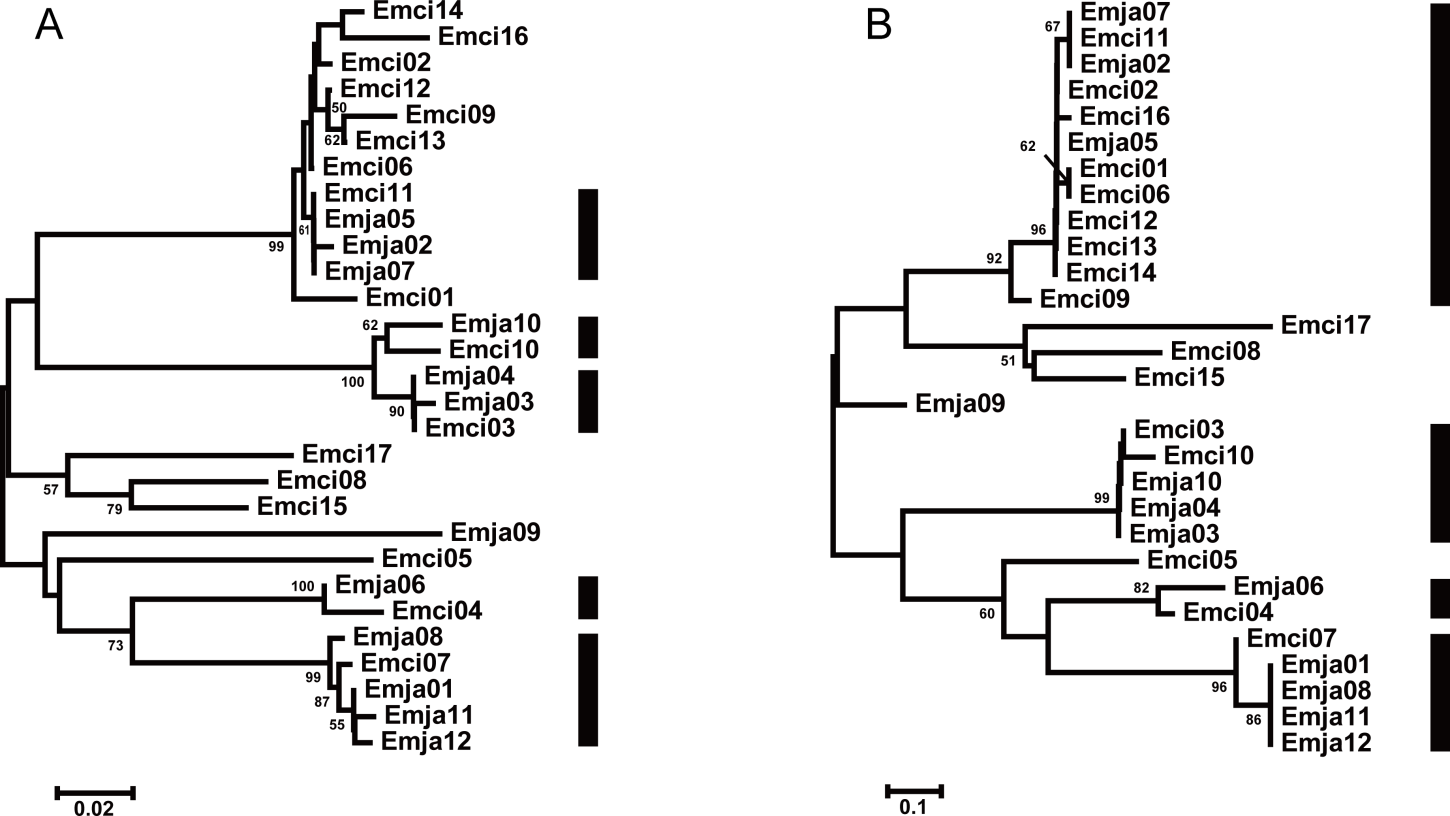
Neighbor joining trees of all MHCIIB exon 2 variants in *Emberiza jankowskii* and *Emberiza cioides*. (A) Tree constructed by comparing synonymous substitutions/synonymous site from codon positions identified as the peptide binding region (putative non-PBR) in HLA (Tong et al., 2006) (74 codons). (B) Tree constructed by comparing non-synonymous substitutions/nonsynonymous site at remaining (putative PBR) sites (15 codons). Black rectangle indicates supported interspecific clades in (A) and (B). Emja, *Emberiza jankowskii*; Emci, * Emberiza cioides*.

We observed a similar pattern among the three different partitions of exon 2, i.e., interspecific clusters in trees. More interspecific clusters were observed when comparing putatively neutral sites than putatively functional sites (Figs. 4A and 4B). A greater number of interspecific clusters were observed in the trees constructed by the contiguous 270 bp fragment of exon 2 (Fig. 3).

For *E. jankowskii*, six alleles were shared by all three populations (Emja01–04, 06, and 07), and three alleles were shared by GHT and ALH (Emja05, 10, and 12). However, some population-specific alleles were identified. For instance, one allele (Emja08, 10% alleles) was only found in GHT, whereas the others (Emja09 and 11, 18% alleles) were only found in ALH.

For *E. cioides*, six alleles were shared by GHT and BYTL (Emci01, 02, 04, 11, 14, and 15), two alleles were shared by GHT and ALH (Emci03 and 10), and one allele was shared by ALH and BYTL (Emci05). However, five alleles (Emci06–08, 16, and 17, 63% alleles) were only found in ALH, and three alleles (Emci09, 12, and 13, 27% alleles) were only found in  GHT.

## Discussion

Species generally survive by adapting to changes in the environment. High levels of genetic diversity can confer an enhanced adaptive capacity upon a species. In this study, the two *Emberiza* species displayed difference in their nucleotide diversity (0.1865 for *E. jankowskii* and 0.1667 for *E. cioides*). Several studies have reported evidence of up to 5–20 MHC class II loci in various other passerines (Bollmer et al., 2010; Zagalska-Neubauer et al., 2010; Balasubramaniam et al., 2016). Compared to those studies, these two *Emberiza* species exhibit relatively low MHC gene diversity, which could be attributable to the primers (Int1f.7/Int2r.1) used in this study because the primer set was just one of three primer sets used for the Common Yellowthroat (*Geothlypis trichas*) and it amplified just five of the 20 possible loci in this species (Bollmer et al., 2010). Although the primers’ ability to detect loci may vary among birds, we do not anticipate significant variation in primer performance between the two *Emberiza* species in this study and thus the estimates in this study should be comparable. A high level of MHC polymorphism is expected for the common non-endangered species, *E. cioides*, but not for the endangered species, *E.  jankowskii*. However, larger differences were detected in the nucleotide diversity of MHCIIB genes between populations within species than between the two *Emberiza* species (Table 1), suggesting similar MHC diversity. Moreover, the difference in the mean number of alleles per individual (0.14 allele/ind) between the two *Emberiza* species was similar.

MHC variability is believed to measure the ability of individuals to adapt to changing environments (including exposure to continuously evolving pathogens and parasites) within and between populations (Sommer, 2005; Spurgin & Richardson, 2010). The number of alleles per individual has been used to assess the adaptive genetic variation in populations (Luo & Pan, 2013). In the present study, the mean number of alleles per individual and genetic diversities of the three populations for *E. jankowskii* displayed little variation (Table  1), suggesting the abilities of individuals to adapt changing environments are similar. In the two sympatric regions, GHT and ALH, *E. jankowskii* showed different genetic diversities with *E. cioides*. The *E. jankowskii* with higher MHC diversity (mean number of alleles per individual and nucleotide diversity) in GHT (Table 1) might recognize greater diversity of pathogens than *E. cioides* population. However, in ALH, the *E. cioides* might display an increased capacity to adapt to changing environments due to the relatively higher MHC diversity than *E. jankowskii* (Table 1).

The TSP model is valid if one of the following conditions are met: (i) the same alleles are shared between allied species; or (ii) similar alleles from closely related species are clustered into one clade (Klein et al., 1998; Klein, Sato & Nikolaidis, 2007). TSP was detected following the generation of phylogenetic trees. Convergent evolution has also been proposed to explain the occurrence of similar alleles between species (Eimes et al., 2015). In this study, we observed strong interspecific clustering of the MHCIIB variants in these two closely related emberizids; however, the clustering was more inclusive, and better supported, when comparing synonymous substitutions at putatively non-selected sites (five clades, Fig. 4A) than with non-synonymous substitutions at putatively selected sites (four clades, Fig. 4B). This suggests that the pattern observed in the phylogenies is more likely to be due to TSP than convergent evolution. Also, the overlapping distribution was extremely obvious, as indicated by all 12 alleles in *E. jankowskii* clustering with the *E. cioides* alleles (Fig. 4A). The existence of identical or similar alleles also indicates that TSP may occur between the two species. TSP refers to ancestral alleles in the population and species that have been maintained over time (Figueroa, Gunther & Klein, 1988; Lawlor et al., 1988). TSP is a common phenomenon in avian species such as ardeid birds (Li, Zhou & Chen, 2011), owls (Burri et al., 2008), penguins (Kikkawa et al., 2009), and passerines (Arnaiz-Villena et al., 2007; Eimes et al., 2015). In the present study, the results suggest that TSP in *E. jankowskii* and *E. cioides* may explain interspecific allelic similarity.

Positive selection refers to the maintenance of alleles containing beneficial mutations that improve individual fitness. The classical standard tests for selection (Tajima’s D, Fu & Li’s D* and Fu & Li’s F*) showed no deviations from the neutral expectations. Considering the level of variation and the short fragment of the MHCIIB gene, these classical methods for testing selection are not powerful (Anmarkrud et al., 2010). The amino acids in PBR that are involved in antigen recognition apparently play major roles in immunologic function. However, our study indicated that putative non-PBRs showed significant and higher ratios of d_*N*_/d_*S*_ than the putative PBR of *E. jankowskii / E. cioides* (Table 2). High ratios of d_*N*_/d_*S*_ were the result of low rates of d_*S*_ (0.089 in *E. jankowskii*, 0.074 in *E. cioides*), rather than the excess of d_*N*_ at non-PBRs (Table 2), which has also been observed in other passerines (e.g., Miller & Lambert, 2004; Sutton et al., 2013). Very low rates of d_*S*_ at non-PBRs may be the results of codon-usage bias (Yang & Bielawski, 2000). When accounting for codon-usage bias by the method of Yang & Nielsen (2000), the d_*N*_/d_*S*_ ratios was less than 1 in these two *Emberiza* species. This suggested that underestimation of d_*S*_ means overestimation of *ω*, with resulting in large errors in the d_*N*_/d_*S*_ ratio (Yang & Bielawski, 2000). Additionally, as the locations of PBRs in these two *Emberiza* species have been inferred from human data, they may in fact differ in their exact locations. Of the 9 codons in *E. jankowskii* and 17 in *E. cioides* from PAML/SLAC/FEL analyses that exhibited positive selection, 4 and 10, respectively, corresponded to known PBRs in humans (Tong et al., 2006; Fig. 2; Tables 3 and 4), while another 3 and 5, respectively, that fell outside human PBRs overlapped with positively selected codons identified in other avian species (Anmarkrud et al., 2010; Zagalska-Neubauer et al., 2010; Sutton et al., 2013; Borg et al., 2011; Fig. 2; Tables  3 and  4), respectively. Considering the function of PBR, we conclude that the MHCIIB gene identified in both species has undergone pathogen-mediated balancing selection.

Recombination is an important mechanism that influences allelic diversity in MHC class I and II loci (Andersson & Mikko, 1995; Schaschl et al., 2005; Wutzler, Foerster & Kempenaers, 2012). It is likely that the generation of genetic diversity was facilitated by the creation of new alleles. Two recombination patterns have been observed. The first pattern involves exon shuffling with recombination occurring between entire exon regions with breakpoints in intronic regions (Holmes & Parham, 1985). The second recombination pattern involves the exchange of small fragments of MHC class I genes (Hughes, Hughes & Watkins, 1993). Signatures of recombination have been reported in avian lineages (Alcaide et al., 2008; Burri et al., 2008; Anmarkrud et al., 2010), and are expected to have an impact on the evolution of avian MHC variation. In this study, we found no signature of recombination between and within *E. jankowskii* and *E. cioides*. However, recombination events could not be excluded to affect the genetic diversity of MHC genes in the present study due to restricted sample sizes.

Above all, the MHC polymorphism in *E. jankowskii* has been facilitated by TSP and positive selection using PAML/SLAC/FEL analyses in the putative PBR in the evolutionary process. It is possible that the relatively low genetic diversity in the endangered *E. jankowskii* might be due to habitat fragmentation. A previous study suggests that the reasons for the local extinction of *E. jankowskii* include cultivation, forest plantations, and undue grazing practices, all of which have led to the destruction of potentially suitable habitats (Wang, Jiang & Gao, 2010). In the ALH population of *E. jankowskii* and *E. cioides,* the extent of MHC-specific polymorphisms in *E. jankowskii* is less than that observed for *E. cioides*(Table 1), which is consistent with the hypothesis that endangered species display reduced genetic diversity compared to common species. Following analysis of the GHT population of both species, MHC-specific polymorphisms were more prevalent in *E. jankowskii* than in *E. cioides*(Table 1). In summary, the comparative MHC diversity of the two *Emberiza* species were variable in sympatric areas.

Further research on MHC genetic diversity using a larger number of individuals and populations is necessary. Furthermore, the primers used in this study have previously been used to successfully investigate a number of MHC class II loci in the Common Yellowthroat (Bollmer et al., 2010). However it was not yet known how well these primers performed in *E. jankowskii* and *E. cioides* before this study. In addition, the method of traditional cloning & sequencing used in this study exhibits lower sequencing depth than next-generation sequencing technology (NGS), which may have impacted the measured level of MHC diversity in the two *Emberiza* species. Thus, species-specific primers for *Emberiza* and the methods of NGS should be used to investigate the MHC characteristics and associated evolutionary mechanisms in endangered *E. jankowskii* populations in the future.

## Conclusions

The present study demonstrates that genetic diversity at the exon 2 of the MHCIIB genes of the endangered *E. jankowskii* and the more common *E. cioides* populations is similar and not very high. Several alleles were observed to be similar between the species as a result of TSP, which might be contributing to MHC polymorphism. Positive selection was considered to play an important role in the MHC class IIB gene evolution of *E. jankowskii* and *E. cioides. Emberiza jankowskii* possesses relatively low levels of polymorphism in their MHC class II genes and relatively low population sizes have been observed in the field. Thus, conservation strategies should be adopted in order to prevent further habitat fragmentation. Moreover, habitats suitable for avian species are likely to be reduced should practice that incorporate excessive agricultural activity and overgrazing continue. This will likely result in a concomitant reduction in population sizes, not only for the endangered *E. jankowskii*, but also for other sympatric birds.

##  Supplemental Information

10.7717/peerj.2917/supp-1 Supplemental Information 1 Sequences

## Additional Information and Declarations

### Field Study Permissions

The following information was supplied relating to field study approvals (i.e., approving body and any reference numbers): National Animal Research Authority in Northeast Normal University, China (approval number: NENU-20080416).

### DNA Deposition

The following information was supplied regarding the deposition of DNA sequences: Sequences can be found in Supplementary Information 1.

### Data Availability

The following information was supplied regarding data availability: GenBank accession numbers KT751182 –KT751210.
